# Long-term psychosocial functioning after Ilizarov limb lengthening during childhood: 37 patients followed for 2–14 years

**DOI:** 10.3109/17453670903473024

**Published:** 2009-12-04

**Authors:** Judith M Moraal, Alda Elzinga-Plomp, Marian J Jongmans, Peter M van Roermund, Petra E Flikweert, René M Castelein, Gerben Sinnema

**Affiliations:** ^1^Departments of Paediatric Psychology; ^2^Orthopaedics, University Medical Centre Utrecht, “Wilhelmina Children's Hospital”, Utrecht, the Netherlands

## Abstract

**Background and purpose** Few studies have been concerned with the patient's perception of the outcome of limb lengthening. We describe the psychological and social functioning after at least 2 years of follow-up in patients who had had a leg length discrepancy and who had undergone an Ilizarov limb lengthening procedure.

**Patients and methods** Self-esteem and perceived competence were measured in 37 patients (aged 17–30 years) both preoperatively and at a mean follow-up of 7 (2–14) years. At follow-up, health-related quality of life, functioning at school, daily activities, and treatment-related experiences were measured, and also retrospectively for the preoperative period.

**Results** Preoperative and follow-up scores for self-esteem were similar. Overall perceived competence scores at follow-up were comparable to that of a healthy normal population. Patients' perceived athletic competence was lower and their perceived level of behavioral conduct was higher. At follow-up, patients had more positive appraisal of their physical appearance. Most health-related quality of life scores were not significantly different to those of the healthy normal population, apart from a reduced gross motor function, less vitality, and more pain. Patients with a remaining leg length inequality (LLI) of more than 2 cm had lower quality of life scores for gross motor function, sleep, pain, vitality, and depressive feelings.

**Interpretation** At an average of 7 years after an Ilizarov limb lengthening procedure, patients still have physical restraints, but they appear to have normal psychosocial functioning, self-esteem, and perceived competence. These patients have quality of life scores comparable to those of norm groups, apart from a reduced gross motor function, less vitality and more pain. Residual LLI of more than 2 cm remains important even after long-term follow-up; these patients report lower quality of life.

## Introduction

The Ilizarov limb lengthening procedure is well-established in the treatment of limb-length discrepancies. Previous evaluations of outcome after Ilizarov limb lengthening have mainly concentrated on clinical and radiographic results rather than on the overall results of psychological and social functioning (Siffert 1987, Paley 1990, Bell et al. 1992, Bonnard et al. 1993, Young et al. 1994). The Ilizarov limb lengthening procedure is psychologically stressful because of its long duration and common complications, including soft tissue problems, restriction in motion of joints, fracture of the lengthened bone, and (sub)luxation of the knee or hip (Ghoneem et al. 1996). Thus, limb lengthening can have a strong psychological impact on the patient (Hrutkay and Eilert 1990, Ghoneem et al. 1996, Morton 1997, Ramaker et al. 2000, Martin et al. 2003). Several studies have shown that psychological problems that may arise during the procedure resolve without further consequences (Hrutkay and Eilert 1990, Ghoneem et al. 1996, Morton 1997, Ramaker et al. 2000).

Perception of pain, patient satisfaction, and physical appearance are considered important outcomes when assessing the success of an Ilizarov limb lengthening procedure. Some authors have reported on pain perception (Young et al. 1994, McKee et al. 1998), satisfaction (Hrutkay and Eilert 1990, Ghoneem et al. 1996, Ramaker et al. 2000, Martin et al. 2003), and physical appearance (Ghoneem et al. 1996, Martin et al. 2003) shortly after an Ilizarov limb lengthening procedure, but until now there has been a lack of data on long-term follow-up.

An Ilizarov limb lengthening procedure will leave scars on the lengthened limb, and even more so when there are complications. To our knowledge, whether or not young adults elect to have plastic surgery to remove these scars has not been investigated.

Few reports have been published about the subjective improvement in physical skills after an Ilizarov procedure. In a follow-up study by Ramaker et al. (2000), 26 adolescents showed no subjective improvement in physical skills at mean 3 (1.5–5.5) years after the procedure, and almost a quarter of the patients still had complaints about their leg. However, Barker et al. (2004) showed that improvement in physical outcome continued up to, but not beyond, 2 years. A study by McKee et al. (1998) showed an improvement in the general health status compared to preoperative data at 2 years after Ilizarov reconstruction, although postoperative scores still showed deficiencies compared to normal controls.

A remaining leg length inequality (LLI) can influence the quality of life (QoL) of young adults. QoL scores are lower in young adults with a remaining LLI of more than 2 cm (Vitale et al. 2006), but it is unknown whether this lower QoL is a long-lasting effect.

There have been no follow-up studies of psychosocial or physical functioning after an Ilizarov limb lengthening procedure beyond a period of 3 years. Some patients who were included in our study were also included in the study by Ramaker et al. (2000). Our study extends the follow-up period to 7 years, and includes 37 patients instead of the 26 in the study by Ramaker et al (2000). Furthermore, in contrast to the latter study, the present study has a broader outlook on psychosocial functioning and is partly prospective.

## Patients and methods

Between January 1990 and May 2003, we performed Ilizarov limb lengthening on 95 children with a leg length discrepancy. All these children had preoperative medical and psychological screening followed by psychological counseling before surgery. Included in this study were the 45 patients who had completed the procedure more than 2 years previously (measured from the time of frame removal). 2 patients were lost to follow-up because of missing medical records. 4 patients refused to participate in our study because they did not want to be reminded of the period of limb lengthening, or because they were too busy. 2 did not return the questionnaires. Thus, 37 patients were included in the study.

All lengthening procedures were performed by metaphyseal corticotomy and callus distraction (van Roermund 1994) (Table 1). A single surgeon performed all lengthenings. The complications reported during the procedure were a fracture in 7 patients, frequent superficial infections, and deep infections of the pin-site in 9 patients. Flexion contractures were noted in 14 lengthenings. Of the 15 femoral lengthenings, 7 patients developed a flexion contracture of the knee. Of the patients who underwent a femoral and tibial lengthening (n = 8), 5 developed a flexion contracture of the knee. Of the 21 tibial lengthenings, 2 patients developed a knee flexion contracture. 2 patients, both in the femoral lengthening group, developed a subluxation of the knee during the lengthening procedure. The knees had aplasia of the cruciate ligaments or a hypoplastic femur, and were without good knee stabilization during lengthening. No subluxation in the hip joint occurred.

**Table 1.** Patients' medical data

Population	n = 37 (22 females)	
Cause		
Congenital (n = 22)		
hypoplasia		14
hemiatrophy		4
skeletal dysplasia		1
femur-fibula-ulna syndrome		1
Ollier's disease		2
Acquired (n = 12)			
trauma		7
infection		4
Perthes' disease		1
unknown		3
Age at time of lengthening (years)	13.2 (8.0–20)	
Number of lengthening procedures		
Patients with 1 lengthening procedure (n = 30)		
femur		10
tibia		14
both segments		6
Patients with 2 lengthening procedures (n = 7)		
2 × tibia		3
2 × femur		2
* 1 × femur, 1 × both segments		1
1 × tibia, 1 × both segments		1
Lengthening		
femoral		15
tibial		21
femoral and tibial lengthening		8
Months in frame **^a^**	7.4 (3.0–12)	
Leg-length discrepancy (cm) **^a^**,**^c^**	5.2 (1.5–16)	
Amount of lengthening (cm) **^a^**	4.1 (1.5–9)	
Months follow-up **^a^**	83 (24–169)	
Age at time of follow-up (years) **^b^**	22 [3.3] (17–30)	
Mean remaining LLI at follow-up ^b^,**^c^**	1.9 [4.1] (0–20)	
LLI equalized		14
LLI ≤ 2 cm		17
LLI > 2 cm		6

**^a^** Mean (range). **^b^** Mean [SD] (range). **^c^** Due to a greater growth velocity in the healthy limb, in one patient the LLI was 16 cm before and after surgery, and 20 cm at the end of the procedure.

At the time of the study, 15 patients had a full-time job, 21 patients were still at school, and 1 patient was unemployed. 20 participants lived with their parents.

### Self-esteem

In the psychological assessment before the procedure and at follow-up, we used the Rosenberg self-esteem scale (RSE) to measure self-esteem (Rosenberg 1965). This questionnaire can be used for children and adults. The RSE is a 10-item self-reported scale with a 4-point Likert scale (Byrne 1996). The total score is derived from a simple summation of the 10-item response values. Higher scores indicate higher self-esteem. Psychometric qualities of the RSE are good (reliability ranges between 0.85 and 0.93) (Rosenberg 1979, Byrne 1996).

### Perceived competence

To investigate perceived competence, we used 2 measures. First, the Dutch version of the Self-Perception Profile for Children (CBSK) (Veerman et al. 1997) was used to measure perceived competence for children aged 8–12 years. It measures perceived competence in 6 categories: scholastic competence, social acceptance, athletic competence, physical appearance, behavioral conduct, and global self-worth. Scores are expressed as percentiles on a scale from 0 to 100. Validity and reliability are satisfactory (Everts et al. 2004). Secondly, the Dutch version of the Self-Perception Profile for Adolescents (CBSA) (Harter 1988) was used to measure self-esteem in children aged 13 years and older. It measures self-esteem in the same categories as described in the CBSK, with the added category of “close friendship”. Validity and reliability are also satisfactory (Everts et al. 2004). The CBSK or CBSA were used preoperatively, while CBSA was used in the follow-up study.

### Health-related quality of life (HRQoL)

To investigate health-related quality of life at follow-up, we used the TNO-AZL Adult Quality of Life questionnaire TAAQOL (Theunissen et al. 1998), which measures a patient's evaluation of his/her own functioning. The questionnaire consists of 45 questions divided into 12 categories. Each category contains 2–4 questions (the actual number per category is given in parentheses): gross motor functioning (4), fine motor functioning (4), pain (4), sleep (4), cognitive functioning (4), social functioning (4), daily activities (4), sexual activity (2), vitality (4), happiness (4), depressive feelings (4), and aggressiveness (3). For each item, the frequency of occurrence of a health status problem is assessed. If such a problem is reported, the emotional reaction to this problem is also determined. The reference period is formulated as “in the last month”. Scores of each subscale are linearly transformed to a scale ranging from 0 to 100, with higher scores indicating better quality of life. The validity of the TAAQOL is satisfactory (Bruil et al. 2004). Cronbach's alpha varies between 0.72 and 0.90, levels that are deemed sufficient to justify the use of the TAAQOL for studies on groups of patients (Bruil et al. 2004).

### Treatment-related experiences

In addition, a specific questionnaire was developed concerning the current functioning related to the leg length discrepancy and satisfaction with the result of the procedure. Patients were asked whether they had physical or psychological complaints at follow-up, and whether they thought those problems were related to the lengthened leg. They were also asked to mention whether the lengthened leg had a negative impact on their “self-confidence”, “temper”, “total appearance”, “athletic performance”, and on the way they lived their lives in general. Patients answered these latter questions for the actual situation at follow-up, and also in retrospect for the period before the limb lengthening procedure. Furthermore, satisfaction was evaluated by means of a rating for the total result on a 10-point scale (the higher the rating, the higher the satisfaction) and patients were asked whether they would consider having the same treatment again if it was indicated. Satisfaction about the appearance of the lengthened leg was measured on the 5-point scale “ugly”, “not very beautiful”, “neutral”, “pretty” and “very beautiful”. Whether patients had current complaints about the leg was evaluated on a 4-point scale categorized as “severe”, “average”, “few”, and “not at all”. Patients answered these same questions in retrospect for the period before the limb lengthening procedure. A visual analog scale (VAS) was used to measure pain at the time of follow-up (the higher the score, the more the pain). Finally, results of plastic surgery were evaluated by means of a rating for the total result on a 10-point scale (the higher the rating, the higher the satisfaction).

In the pre-procedural psychological assessment, 10 patients had completed the RSE, 13 patients had completed the Self-Perception Profile for Children (CBSK), and 17 patients had completed the Self-Perception Profile for Adolescents (CBSA). This resulted in a preoperative measurement of all categories of perceived competence in 30 cases, and for the category concerning “close friendship” in 17 cases. At follow-up, the RSE, the CBSA, the TAAQOL, and the additional questionnaire concerning treatment-related experiences were completed by all respondents. All questionnaires were sent and returned by mail.

### Statistics

The data were analyzed using SPSS software version 12.01. To compare pre- and postoperative means of self-esteem, the Wilcoxon signed-ranks test was used. Perceived competence in 30 patients for whom pre-and postoperative data were available was compared using paired t-tests. One-sample t-tests were performed to test the differences between patients in our study and published Dutch norms on the TAAQOL questionnaires. To investigate the association between self-esteem, perceived competence, health-related quality of life, and treatment-related experiences on the one hand, and the number of limb lengthening procedures (1, n = 30; 2, n = 7), etiology of LLI (congenital, n = 22; other, n = 15) and remaining LLI (less than 2 cm, n = 31; more than 2 cm, n = 6) on the other, either chi-square or Student's t-tests were performed. The significance level for the analyses was set at 0.05.

### Ethical considerations

Approval from the medical ethical committee at University Medical Centre Utrecht, “Wilhelmina Children's Hospital”, was obtained for this study (protocol number 04/107-K). Informed consent was obtained from all patients.

## Results

### Self-esteem

The mean total score of RSE self-esteem for 37 patients at follow-up was 34 (SD 5.9) (20–40). For the 10 patients for whom preoperative data on self-esteem were available, the mean total RSE score in the preoperative period (34 (SD 6.9) (20–40)) was similar to that in the postoperative period (33 (SD 6.4) (20–40)) (p = 0.5). The differences in mean total RSE self-esteem score of 32 for the 6 patients who had a remaining LLI of more than 2 cm and of 35 for the 31 patients who had a remaining LLI of less than 2 cm did not reach statistical significance (p = 0.3).

### Perceived competence

At follow-up, most perceived competence scores fell in the average range (between the thirty-fifth and sixty-fifth percentiles), except for “athletic competence” and “behavioral conduct”. The mean percentile score of 35 for “athletic competence” (SD 29) fell within the low perceived competence range while the mean percentile score of 65 for “behavioral conduct” (SD 29) fell within the high range (n = 37). Data on perceived competence at follow-up compared to the preoperative data were available for 30 patients who completed the CBSK or CBSA before and after the procedure (Table 2). Their mean overall percentile scores (52 (SD 22)) were similar to that of the total group of 37 patients at follow-up (53 (SD 20)) (p = 0.4). In these 30 patients, an improvement was seen in the follow-up score for the category “physical appearance” (p = 0.02). We analyzed various subgroups to determine whether there were any factors influencing perceived competence in this patient group. Results of univariate analysis revealed a main effect of remaining LLI of more than 2 cm on “physical appearance” (p < 0.01) and “global self-worth” (p < 0.01). In these scores, patients with an LLI of more than 2 cm scored statistically significantly lower. Whether the patients had had 1 or 2 lengthening procedures and also the etiology of LLI (congenital, n = 22; other, n = 15), had no statistically significant effects on perceived competence at follow-up (p = 0.2 and p = 0.1, respectively).

**Table 2.** Mean scores and standard deviations of perceived competence (range 0–100) before the procedure and at follow-up. High scores indicate higher perceived competence

Scale	Before procedure	SD	At follow-up	SD	Mean difference	95% CI	SD	p-value
Scholastic competence (SC) **^a^**	55	30	65	31	11	-5–26	41	0.2
Social acceptance (SA) **^a^**	56	31	49	34	-7	-21–7	37	0.3
Athletic competence (AC) **^a^**	42	28	36	30	-6	-18–5	31	0.3
Physical appearance (PA) **^a^**	49	34	63	31	15	3–26	31	0.02 **^c^**
Behavioral conduct (BC) **^a^**	55	27	67	30	12	-2–26	36	0.08
Close friendship (CF) **^b^**	51	26	48	33	-3	-20–15	34	0.7
Global self-worth (GS) **^a^**	59	33	60	33	1	-10–12	30	0.9
Overall (OV) **^a^**	52	22	56	21	4	-4–11	20	0.3

**^a^** n = 30 **^b^** n = 17 **^c^** p < 0.05. Paired t-test.

### HRQoL

The TAAQOL scores (Table 3) were compared to data derived from a control group consisting of 428 healthy adults aged 18–30 years. This group had similar sex distribution and a mean age similar to that of the patient population. The TAAQOL scores of patients were lower than in the general healthy population in the categories “gross motor” (walking upstairs, bending over, walking 500 yards) (p < 0.001), “pain” (pain in the extremities, pain in muscles) (p < 0.001), and “vitality” (an energetic feeling about doing things in daily life) (p = 0.05) (Table 3). After dividing the patients into 2 groups, using the 2 cm cutoff value, patients with an LLI of > 2 cm scored lower in many more domains than patients with an LLI of ≤ 2 cm (Table 4). QoL scores were lower in patients with an LLI of > 2 cm in the domains “gross motor functioning” (p < 0.01), “sleep” (p = 0.03), and “depressive feelings” (p < 0.01) (Table 5). Whether or not the patients had had 1 or 2 limb lengthening procedures had no effect on overall HRQoL at follow-up (p = 0.7). Furthermore, the etiology of LLI had no statistically significant effect on overall HRQoL at follow-up (p = 0.08).

**Table 3.** Mean HRQoL scores (TAAQOL; range 0–100) for young adults after an Ilizarov limb lengthening procedure and for the general population. High scores indicate better quality of life

Scale	Mean for Ilizarov patients (n = 37)	SD	Mean for general population (n = 422–428)	SD	Mean difference	95% CI	p-value
Gross motor	77	26	97	10	20	11–29	< 0.01 **^a^**
Fine motor	99	7	99	4	1	-1–2	0.34
Cognitive function	86	20	89	18	3	-3–10	0.30
Sleeping	73	29	82	21	9	-1–19	0.08
Pain	65	26	84	17	20	10–29	< 0.01 **^a^**
Social function	92	14	91	15	-1	-6–4	0.78
Daily activities	85	22	89	18	4	-3–10	0.25
Sexuality	93	16	90	20	-4	-10–3	0.32
Vitality	64	24	72	20	4	0–16	0.05 **^a^**
Happiness	72	21	75	18	3	-4–9	0.39
Depressive feelings	82	18	84	16	3	-3–8	0.43
Aggressiveness	91	13	89	15	3	-3–7	0.43

Student's t-test. **^a^** p < 0.05.

**Table 4.** Mean HRQoL domain scores (max. range 0–100)

Scale	Normal group (n = 422–428)	All (n = 37)	LLI of ≤ 2 cm (n = 31)	LLI of > 2 cm (n = 6)
Gross motor	97	77 **^a^**	83 **^a^**	49 **^a^**
Fine motor	99	99	99	94
Cognitive function	89	86	90	66
Sleep	82	74	77	49 **^a^**
Pain	84	65 **^a^**	68 **^a^**	47 **^a^**
Social function	91	92	93	83
Daily activities	89	85	90	60
Sexuality	90	93	94	88
Vitality	72	64 **^a^**	66	51 **^a^**
Happiness	75	72	74	65
Depressive feelings	84	82	86	58 **^a^**
Aggressiveness	89	91	92	87

**^a^** p < 0.05. Student's t-test.

**Table 5.** Mean HRQoL domain scores (max. range 0–100) according to severity of LLI

Scale	LLI of ≤ 2 cm (n = 31)	LLI of > 2 cm (n = 6)	Mean difference	95% CI	p-value
Gross motor	83	49	-34	-55 to 12	< 0.01 **^a^**
Fine motor	99	94	-6	-22 to 10	0.4
Cognitive function	90	66	-25	-60 to 11	0.2
Sleep	77	49	-29	-53 to -4	0.03 **^a^**
Pain	68	47	-21	-44 to 2	0.07
Social function	93	83	-11	-43 to 21	0.4
Daily activities	90	60	-30	-68 to 9	0.1
Sexuality	94	88	-7	-41 to 28	0.6
Vitality	66	51	-15	-36 to 7	0.2
Happiness	74	65	-8	-28 to 11	0.4
Depressive feelings	86	58	-28	-41 to -14	< 0.01 **^a^**
Aggressiveness	92	87	-8	-27 to 11	0.4

**^a^** p < 0.05. Student's t-test.

### Treatment-related experiences

At follow-up, 21 patients had physical complaints. In 11 of them, these physical complaints were considered to be related to the lengthening of the leg. 5 patients reported psychological complaints; 3 of them reported that these problems were related to the lengthened leg. According to the outcome of the additional questionnaire, after the procedure the impact of the lengthened leg diminished in all domains of psychosocial functioning. Looking back at the period before the Ilizarov procedure, 15 patients stated at follow-up that the affected leg had had a negative effect on their self-confidence, while 9 patients stated that the lengthened leg still had a negative effect on their self-confidence. The impact of the lengthened leg on the remaining domains also diminished at follow-up: temper (9 before and 4 at follow-up), total appearance (18 before and 12 at follow-up), athletic performance (23 before and 18 at follow-up), and the way they lived their lives in general (9 before and 5 at follow-up).

### Satisfaction

Mean rating of the overall results of the total Ilizarov procedure was 7 (SD 1.7) (2–9). At follow-up, 29 patients stated that they would have the same treatment again if it was indicated. Appraisal of the lengthened leg at follow-up (“ugly”, 8; “not very beautiful”, 11; “neutral”, 13; “pretty”, 2; “very beautiful”, 3; “don't know”, 0) differed little from their retrospective appraisal (“ugly”, 6; “not very beautiful”, 13; “neutral”, 16; “pretty”, 1; “very beautiful”, 0; “don't know”, 1). Before the procedure, 12 patients had “severe to average complaints” about the shorter leg and 25 patients stated that they had “few or no complaints at all”. After the procedure, complaints were “severe to average” in 9 patients, while 28 patients stated that they had “few or no complaints at all”. Patients who had a remaining LLI of 2 cm or less were more satisfied, as measured by the grade given to the results of the procedure (mean 7.6), than patients who had a remaining LLI of more than 2 cm (mean 5.1) (p = 0.02).

### Pain

21 patients reported pain in the lengthened leg at the time of follow-up. Mean VAS score for pain at follow-up was 2.2 (SD 2.8) (0–8.6). Patients who had a remaining LLI of more than 2 cm experienced significantly more pain in the lengthened leg (mean score 5.0 (SD 3.0)) than patients who had a remaining LLI of 2 cm or less (mean score 1.2 (SD 2.5)) (p = 0.01).

### Plastic surgery

11 patients had gone to a plastic surgeon for surgical removal of scars; 2 of them had such a procedure planned for a second time. The mean score for the result of the plastic surgery was 5.6 (SD 1.3) (3–7). 1 patient had plastic surgery planned. 6 patients had not undergone plastic surgery but were interested; 19 patients did not have plastic surgery and were not interested.

## Discussion

Although some psychosocial follow-up studies on the Ilizarov limb lengthening procedure have been published, our study is the first to take a long-term point of view: the mean period of follow-up was 7 (2–14 years). Furthermore, we used standardized questionnaires concerning a wide range of domains of psychosocial functioning. This way of testing health status, or HRQoL, is similar to the methodology used by McKee et al. (1998) and Vitale et al. (2006). Our study on 37 young adults was partly prospective, which contrasts with other studies that have been exclusively retrospective (Hrutkay and Eilert 1990, Ghoneem et al. 1996, Ramaker et al. 2000).

The orthopedic results of the procedure in our patients were similar to those of many recent reports on the treatment of length discrepancy of the lower extremity in terms of average leg length inequality (LLI), average gain in length, average residual limb length discrepancy, cause of the leg length inequality, and the duration of the initial limb lengthening procedure (Siffert 1987, Paley 1990, Bell et al. 1992, Bonnard et al. 1993, Young et al. 1994).

Improvement in physical functioning over time after an Ilizarov limb lengthening procedure was described by Barker et al. (2004); recovery was slowest at the early stages after removal of the frame and greatest between 6 months and 1 year; improvement continued up to, but not beyond, 2 years. Ramaker et al. (2000) concluded from their study that there are no short-term physical benefits of the limb lengthening procedure, and almost a quarter of the children still had complaints about their leg. These authors stated that the rationale of the procedure is to prevent handicaps in the long run. We found that at an average of 7 years after the lengthening procedure, 9 of 37 patients still had moderate to severe complaints about the lengthened leg. However, compared to the preoperative period, patients reported fewer complaints. Also, the negative effect of the affected leg on their daily functioning was reduced in all domains after the limb lengthening procedure (self confidence, temper, physical appearance, and athletic functioning).

Assessment of pain perception by an HRQoL questionnaire in our study showed similar findings to a study by McKee et al. (1998). There was a reduction in pain compared to preoperative scores, but individuals still experienced more pain than control populations. Although 21 of our patients still reported pain, the intensity of the pain was not very high (an average VAS score of 2.2).

Satisfaction of the patient is an important outcome when assessing the success of the procedure. Our patients had a positive attitude; the mean score for satisfaction about the result was 7 (2–9) (max. range 1–10), and compared to the preoperative period patients were more content with their lengthened leg. At follow-up, 29 patients stated that they would have the same treatment again if it was indicated. These findings are similar to those for the patients of Hrutkay & Eilert (1990), Ghoneem et al. (1996), and Ramaker et al. (2000).

There have been no previous studies concerning self-esteem and perceived competence of patients in this population. Our study shows that self-esteem and perceived competence can be quite sufficient and within normal limits even a long time after undergoing the Ilizarov procedure. Furthermore, measurements of perceived competence showed an increase in positive appraisal of physical appearance relative to preoperative data. Ghoneem et al. (1996) also measured positive appraisal of physical appearance in their patient group; 34 of 45 patients who had had reconstruction of deformities of the lower extremities with the Ilizarov method were satisfied with their appearance.

Hrutkay and Eilert (1990), Ghoneem et al. (1996), and Ramaker et al. (2000) reported normal psychological functioning at 3.5 years after the procedure. McKee et al. (1998) used standardized health-status outcome assessments, as used in our study, and reported lower HRQoL than the normal population, but scores were higher than preoperative scores. One of the important findings of our study is that at an average of 7 years after the Ilizarov limb lengthening procedure, except for pain, gross motor function and vitality, HRQoL was not statistically significantly different from that of a healthy normal population.

In the study by Ramaker et al. (2000), there was no relationship between the final, overall judgment of the result after the Ilizarov limb lengthening procedure and the LLI that remained. Our results, however, show that long after the procedure, patients who had a remaining LLI of more than 2 cm were less satisfied. Also, measures of perceived competence and HRQoL were lower for patients with an LLI of > 2 cm. This is similar to findings of Vitale et al. (2006). One explanation for the finding that patients with an LLI of > 2 cm have lower scores on self-esteem, perceived competence, and HRQoL may be that these patients encounter more limitations in daily activities, such as restrictions in sporting and dependency on orthoses. In addition, when a LLI is visible because of orthoses, reactions from other people might influence the psychological functioning of these patients.

Our findings will be useful regarding the preoperative and postoperative phases of the Ilizarov limb lengthening procedure. Parents and children who have such a procedure planned can be informed that the procedure involves a stressful and painful period, but that the prospect will be positive in the long run if the discrepancy in leg length is reduced to less than 2 cm. Adequate psychosocial functioning can be expected following Ilizarov limb lengthening if the discrepancy is reduced to less than 2 cm, if chronic pain is not present, and if the procedure has been successful from the point of view of technical outcome and physical function.
